# Integrated Nutrient Management Enhances the Growth and Yield of Cauliflower (*Brassica oleracea* var. *botrytis*) in Calcareous Soil: A Field Experiment with Mineral, Organic, and Biofertilizers

**DOI:** 10.1021/acsomega.5c09287

**Published:** 2025-12-12

**Authors:** Mohammed Ranjous, Akram Al-Balkhi, Areej Al-Khader, Abd Al Karim Jaafar, Andrés Rodríguez-Seijo

**Affiliations:** † Department of Soil Science, Faculty of Agricultural Engineering, 108042 University of Damascus, Damascus 30621, Syria; ‡ General Commission for Scientific Agricultural Research (GCSAR), Damascus 30621, Syria; § Departamento de Bioloxía Vexetal e Ciencias do Solo, Área de Edafoloxía e Química Agrícola. Facultade de Ciencias de Ourense, 16784 Universidade de Vigo, As Lagoas s/n, Ourense 32004, Spain; ∥ Instituto de Agroecoloxía e Alimentación (IAA), Universidade de Vigo, Campus Auga, Ourense 32004, Spain

## Abstract

Calcareous soil, which covers large areas of Syria and other arid regions, is characterized by high pH, elevated calcium carbonate content, and low availability of essential nutrients, posing significant constraints to vegetable production. Although integrated nutrient management combining mineral, organic, and biofertilizers has shown promise in improving crop performance, field-based evidence from calcareous soil in Syria remains limited. This study aimed to identify the most efficient fertilization strategy for cauliflower (*Brassica oleracea* L. var. *botrytis*) in calcareous soil. We hypothesized that a balanced mixture of mineral, organic, and biofertilizers would enhance nutrient availability and crop performance more effectively than single-fertilizer applications. For this, a field experiment was conducted during the 2024–2025 growing season (October–February) at the Shabaa Research Station using a randomized complete block design with 13 fertilization treatments and three replicates. At the end of the experimental period, soil and plant macronutrients and cauliflower growth parameters were measured. Results demonstrated that the treatment combining 50% mineral fertilizer, 25% fermented cow manure, and 25% biofertilizer significantly (*p* <0.05) enhanced the plant height (51.0 cm), head diameter (31.24 cm), and total yield (43.35 tons ha^–1^), representing a 44.5% increase over the unfertilized control and outperforming mineral fertilization alone. Treatments with a high share of mineral fertilizers (75% mineral fertilizer + 25% organic fertilizer) increased the plant nutrient content but were less effective than balanced mixtures, in terms of crop yield. Moreover, individual fertilizer application resulted in a significant reduction in plant growth compared to the use of mixtures. This study highlights the agronomic and environmental benefits of integrated fertilization strategies in nutrient-deficient calcareous soil, including improved nutrient use efficiency, reduced chemical fertilizer input, and enhanced soil properties.

## Graphical abstract

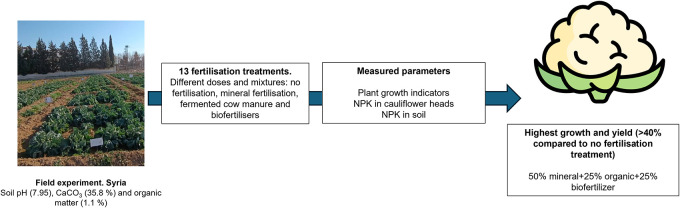

## 1 Introduction

Cauliflower (*Brassica oleracea* L. var. *botrytis*) is a significant cash crop and is one of the most widely consumed vegetables in the world, due to its nutritional value (i.e., high fiber content, low sodium content, and calories) and medicinal properties, including phytochemicals, which offer potential protection against certain diseases.^1–3^ However, its successful cultivation depends on adequate nutrient supply—particularly nitrogen (N)—as well as soil structure and pH (optimal range: 6.5–7.5).^3–5^ In highly acidic or alkaline soils, nutrient imbalance becomes exacerbated, reducing the yield and crop quality and leading to economic losses.^2,3,5–7,9^

In general, mineral fertilizers supply essential nutrients and can boost short-term growth. However, their overuse poses risks such as nutrient imbalance, dependency, and environmental impacts on soils and surrounding waters.^10^ Organic amendments can improve the soil structure, increase the organic carbon content, enhance water retention, and supply slow-release nutrients.^2,5,8,11^ Biofertilizers (i.e., nitrogen-fixing bacteria, phosphate-solubilizing microorganisms, and/or mycorrhizal fungi) can enhance nutrient mobilization and rhizosphere activity,^12,13^ offering complementary benefits when combined with mineral or organic sources and achieving yields comparable to those of mineral fertilization.^2,14^

Integrated nutrient management (INM), which combines mineral, organic, and biological fertilization, has been widely recognized as an effective strategy to balance immediate and long-term nutrient supply. Different studies^2,3,15^ reported significant improvements in cauliflower yield and nutrient uptake with INM compared to individual-source fertilization.^5^ For example, field experiments in Nepal with mineral, organic, and biofertilizers showed that integrated applications, e.g., half the recommended dose of NPK + vermicompost + biofertilizers (*Azospirillium* and vesicular arbuscular mycorrhiza) have achieved the best results with the highest plant height, root structure, yield, and net return compared to different single and mixed fertilizer applications.^15^ Similar results were also reported by authors^2,3,16,17^ with increases in nutritional parameters and crop yield when combining mineral fertilizers with organic and/or bio-organic fertilizers.^2^

The relevance of INM is particularly high in calcareous soil, which dominates agricultural areas in Syria and cover about 30% of Earth’s surface, especially in arid and semiarid regions. Calcareous soil contains high levels of calcium carbonate (CaCO_3_), which can limit phosphorus availability by fixing phosphorus with calcium ions, which tend to become immobilized at alkaline pH.^18^ In general, this soil has a low-nutrient availability for crops, particularly P, Cu, Zn, and Fe, with significant impacts on crop production.^18,19^ In this sense, organic amendments can help mitigate these issues by improving soil porosity, microbial biomass, and water retention and by releasing organic acids during decomposition that enhance micronutrient solubilization (particularly Fe and Zn).^19–22^ Moreover, biofertilizers can act synergistically with organic and mineral sources. Their application enhances rhizosphere activity and facilitates nutrient mobilization processes, particularly in environments with low organic matter and high pH.^12,13,23^

Therefore, supplementing mineral fertilizers with organic and biofertilizers can alleviate these constraints by modifying the soil pH, enhancing the microbial activity and phosphate solubilization, increasing the cation exchange capacity, and improving the micronutrient availability.^24–26^ Despite the benefits of INM in different cropping systems, evidence for Syrian soils is limited.^15,25,26^ Studies have focused on mineral or organic fertilization alone, with scarce information on combinations of different types of fertilizers.

In this study, we hypothesize that partial substitution of mineral fertilizers with organic and biofertilizers improves the yield and soil fertility without reducing productivity. To test this hypothesis, a field experiment was conducted using different fertilization treatments (individual applications of mineral, organic, and biofertilizers, and combinations of them). Plant growth, yield, and soil physicochemical properties were assessed to evaluate how each fertilization strategy performs under specific conditions of calcareous soil in Syria.

## 2 Methodology

### 2.1 Experimental Area

The field experiment was conducted during the 2024–2025 growing season (from October 15, 2024 to February 17, 2025) at the Shabaa Research Station, which is affiliated with the General Commission for Scientific Agricultural Research (GCSAR). The station is located in the Rif Dimashq Governorate, Syria (33.424549°N, 36.403005°E; 639 m above sea level). The region experiences a Mediterranean semiarid climate, characterized by cold, wet winters and hot, dry summers. The average annual precipitation is approximately 200 mm, predominantly occurring between November and April. During the experimental period, the total rainfall recorded at the nearest meteorological station (Dawar Al-Matar, ∼15 km away) was 115.5 mm, which is distributed as follows: 1 mm in October, 26 mm in November, 26 mm in December, 36.2 mm in January, and 26.3 mm in February. The average monthly temperatures ranged from 5.2 °C in January to 14.8 °C in October.

### 2.2 Crop Selection

*Brassica oleracea* is cultivated worldwide as a vegetable crop, and its various forms are generally recognized as varieties instead of subspecies.^1,2^ Cauliflower seedlings (*Brassica oleracea* L. var. *botrytis*, cv. “Casper” supplied by a Syrian seed company) were manually transplanted on October 15, 2024. The planting spacing was 50 cm between rows and 50 cm between plants within a row, resulting in a plant density of 4 plants per row.

### 2.3 Soil Analysis

Soil sampling of each subplot was conducted at the beginning and end of the field experiment. Soil samples from each subplot treated with different fertilizers were characterized at the beginning and end of the field trial.

Soil texture was assessed using the hydrometer method, with sodium hexametaphosphate employed as the dispersing solution.^27^ Soil pH and electrical conductivity were measured in deionized water (in 1:2.5 and 1:5 soil-to-water extracts, respectively).^28,29^ Total nitrogen (N) was determined using the Kjeldahl digestion method, available phosphorus (P) was measured using the Olsen method and spectrophotometric data,^30^ while the available potassium (K) was extracted using ammonium acetate and measured with a flame photometer.^31^ Nitrogen was extracted by KCl extraction.^32^ The total calcium carbonate (CaCO_3_) content was determined by titration with HCl and back-titration using sodium hydroxide.^31^ Organic matter and organic carbon content were estimated using the Walkley–Black wet oxidation method.^33^

Table 1 presents the soil analysis results before the plant field experiment. The soil has a clay texture with an alkaline pH, reduced salinity, high calcium carbonate and available phosphorus contents, a moderate amount of mineral nitrogen, but low levels of organic matter, typical of arid or semiarid areas.^34^ The soil is classified as clay according to the USDA textural triangle, and as calcaric vertisol under the World Reference Base (WRB) system, due to its high clay content (>30%), presence of calcium carbonate (>15%), and shrink–swell properties typical of vertisol in semiarid Syria.^19^

**Table 1:** Soil Analysis before Soil Planting^a^

parameter	units	value
sand	%	26
silt	%	21
clay	%	53
soil texture (USDA classification)		clay
soil pH_H_2_O(1:2.5)_		7.95
soil EC_1:5_	dS m^–1^	0.72
total CaCO_3_	%	35.8
organic matter	%	1.1
total nitrogen	%	0.06
mineral N	mg kg^–1^	11.6
available K	mg kg^–1^	300
available P	mg kg^–1^	16.8

a Average values (*n* = 3).

### 2.4 Mineral, Organic, and Biofertilizer Selection

Mineral fertilizers, supplying nitrogen (N), phosphorus (P), and potassium (K), were used. The standard recommended dose (considered 100%) for mineral fertilizers was 264 kg ha^–1^ urea (46% N), 33 kg ha^–1^ triple superphosphate (46% P_2_O_5_), and 40 kg ha^–1^ potassium sulfate (50% K_2_O).

Fermented cow manure (FCM) was applied as the organic fertilizer at a standard application rate of 12,000 kg ha^–1^. The FCM was characterized prior to the application. The pH and EC were measured in a 1:10 dilution of the fertilizer in water. The total nitrogen was determined using the Kjeldahl method. The total phosphorus (P) was determined via wet digestion using a mixture of nitric and perchloric acids and measured with a spectrophotometer.^35^ The organic matter was determined by the loss-on-ignition method at 600 °C,^35^ while the organic carbon was estimated via dichromate oxidation.^33^ The FCM was characterized prior to application and contained 2% total N, 1.2% P_2_O_5_, 1.8% K_2_O, and 35% organic matter and had a C/N ratio of 20.7.

To ensure nitrogen equivalence across treatments, the nitrogen contribution from FCM was calculated and balanced against urea application in the combined treatments. For example, in a treatment with 50% mineral N and 50% organic N, the amount of urea was reduced proportionally, and the remaining N requirement was supplied by the calculated amount of FCM. This approach allowed for a fair comparison of the fertilization strategies based on their nitrogen supply potential. The application rates of each fertilizer are summarized in Table 2.

**Table 2:** Application Rates of Mineral, Organic, and Biofertilizers Used in the Experiment

	mineral fertilizer				organic fertilizer		biofertilizer	
treatment	%	urea (kg ha^–1^)	triple superphosphate (kg ha^–1^)	potassium sulfate (kg ha^–1^)	%	kg ha^–1^	%	L ha^–1^
T0	no fertilization							
T1	100%	264	33	40				
T2					100%	12,000		
T3							100%	7.5
T4	50%	132	16.5	20	50%	6000		
T5	50%	132	16.5	20			50%	3.75
T6	75%	198	24.75	30	25%	3000		
T7	75%	198	24.75	30			25%	1.87
T8	25%	66	8.25	10	75%	9000		
T9	25%	66	8.25	10			75%	5.62
T10					50%	6000	50%	3.75
T11	50%	132	16.5	20	25%	3000	25%	1.87
T12	33%	87.1	10.9	13.2	33%	3960	33%	2.47

The biofertilizer used was a commercially available multistrain microbial inoculant, EM-1 (Effective Microorganisms-1, EMRO, Japan). It contained a consortium of photosynthetic bacteria (*Rhodopseudomonas* spp.), lactic acid bacteria (*Lactobacillus* spp.), actinomycetes (*Streptomyces* spp.), fungi (*Trichoderma* spp.), and yeast (*Saccharomyces* spp.), with a total microbial concentration of 2.5 × 10^8^ CFU mL^–1^. The standard application rate was 7.5 L ha^–1^, which corresponds to the recommended local application rate. The biofertilizer was diluted 1:20 with water and applied together with irrigation as a soil drench during the transplanting stage.

All fertilizers (mineral, organic, and biofertilizers) were applied basally, 1 day before transplanting. The mineral fertilizers and FCM were broadcasted uniformly onto the respective plots and incorporated into the top 15 cm of soil during final land preparation. The diluted biofertilizer solution was applied directly to the planting holes.

### 2.5 Experimental Design

Given that the treatments represent predefined integrated fertilization packages rather than factorial combinations of independent variables, a Randomized Complete Block Design (RCBD) with three replicates was employed to evaluate their comparative efficacy. The experimental area was divided into three blocks, each containing 13 plots, with each plot randomly assigned one of the 13 fertilization treatments. This design was chosen to account for any potential spatial variability in soil properties across the field.

Prior to planting, the experimental field was plowed using a moldboard plow to ensure uniform soil preparation. Afterward, the land was manually divided into plots (8 m^2^) using traditional hand tools to maintain precision and consistent plot size. A safety margin (buffer zone) was maintained between plots to minimize any possible edge effects. Fertilizer quantities—mineral, organic, and biofertilizers—were calculated based on this plot size, considering the preplanting soil analysis results for the 2024–2025 season and according to fertilization recommendation tables issued by the Syrian Ministry of Agriculture. Fertilizer amounts were adjusted based on the specified percentages of the standard recommended dose: 100%, 75%, 50%, 25%, and 33% (Table 2). The treatments of each block are shown in Table 2. This approach aimed at evaluating the crop response to reduced fertilizer inputs under experimental conditions.

Supplementary irrigation was applied eight times using a sprinkler system (approximately every 10–12 days) to maintain optimal soil moisture, as winter rainfall was insufficient. Weeding was performed manually four times during the growing season (at 20-day intervals). No chemical pesticides were applied; regular field monitoring confirmed the absence of significant pest or disease infestations that would impact the yield. The crop was harvested at physiological maturity on February 17, 2025. At the end of the experimental period and after plant samples were harvested, soil samples were collected from the middle of each plot to ensure representative sampling and avoid border effects.

### 2.6 Cauliflower Growth, Yield, and Tissue Analysis

At harvest, the following parameters were recorded from each plot. (i) Plant height (cm): measured from the soil surface to the apex of the canopy; (ii) head diameter (cm): the average diameter of ten randomly selected marketable cauliflower heads per plot; (iii) yield (t ha^–1^): the total weight of all harvestable cauliflower heads from each plot was recorded and converted to tons per hectare.

Samples of cauliflower heads from each plot were oven-dried at 70 °C to a constant weight, ground to a fine powder, and analyzed for nutrient content: (i) total nitrogen (N) using the micro-Kjeldahl digestion method,^36^ (ii) total phosphorus (P) analyzed by wet digestion followed by colorimetric determination using the vanadomolybdate method,^37^ (iii) total potassium (K) measured by flame photometry after wet digestion.^38^

### 2.7 Statistical Analysis

All measurements were performed in triplicate (*n* = 3). Data were subjected to one-way Analysis of Variance (ANOVA) using GenStat software (version 12.1). The assumptions of ANOVA, namely normality of residuals and homogeneity of variances, were verified using the Kolmogorov–Smirnov test and Levene’s test, respectively.^39^ Where the ANOVA indicated significant treatment effects, means were separated using Fisher’s Least Significant Difference (LSD) test at a 5% significance level (*p* <0.05).

## 3 Results and Discussion

### 3.1 Plant Growth Indicators: Plant Height, Head Diameter, and Yield

#### 3.1.1 Plant Height and Head Diameter

The results showed significant differences among the fertilization treatments regarding their effect on cauliflower plant height. Plant heights ranged from 39.75 cm (no fertilization) to 51.00 and 49.85 cm beneath the mixture of all fertilizers under different proportions (Figure 1a). Among other treatments, T6 (75% mineral fertilizer + 25% fermented cow manure), T7 (75% mineral fertilizer + 25% biofertilizer), and T10 (50% biofertilizer + 50% fermented cow manure) recorded heights of 47.23 47.20, and 46.00 cm, respectively, which were significantly higher than individual fertilization incorporation (Figure 1a). Treatments with only organic or biofertilizers achieved similar heights of 43.53 and 43.72 cm, respectively, with no significant difference from treatments T8 and T9, with mixed combinations. This suggests a slower fertilization effect from these sources compared to chemical fertilizers, despite their important role in improving soil fertility over the long-term (Figure 1a).

**Figure 1:**
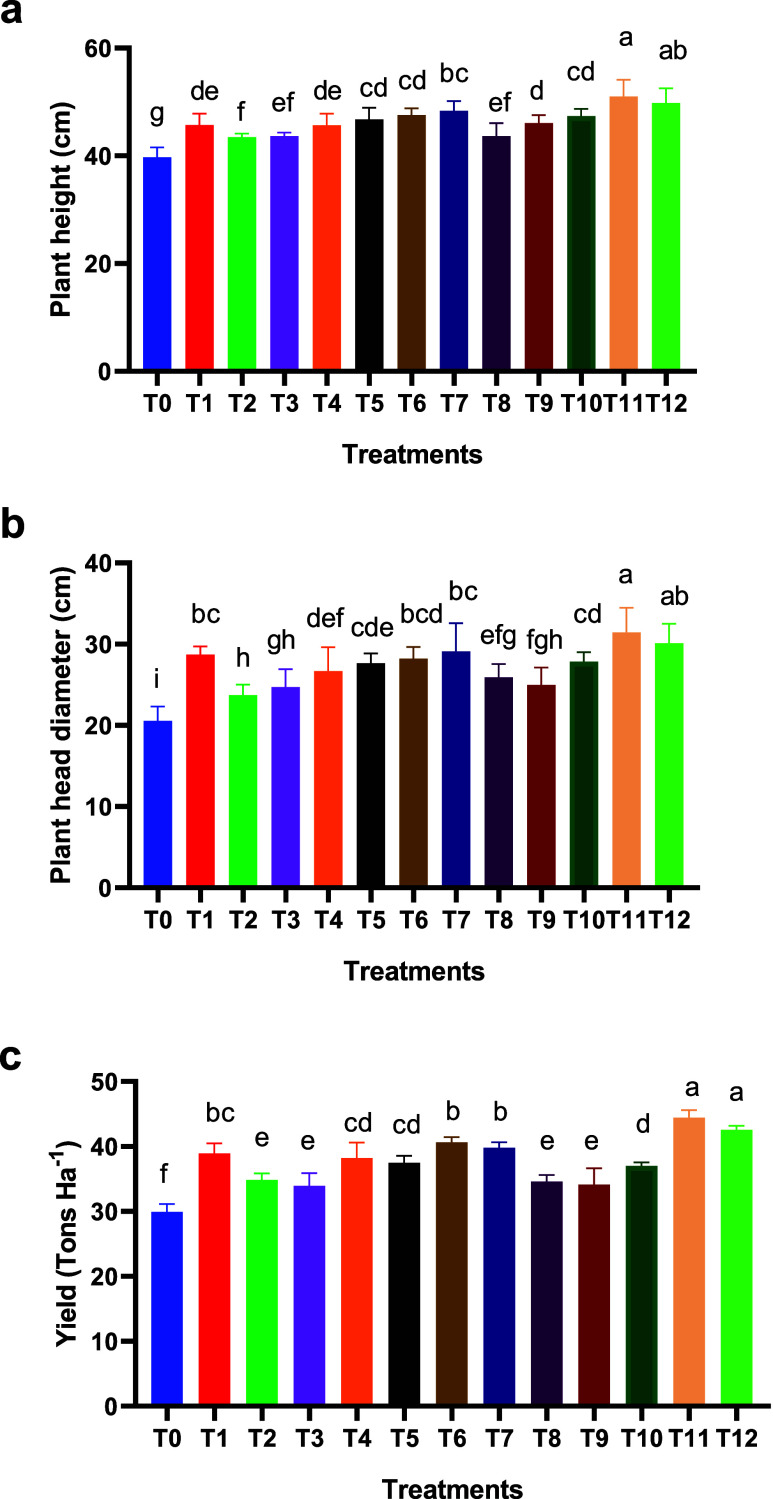
Cauliflower growth parameters in soils treated with different fertilization treatments. Data are represented as mean ± SD (*n* = 3). Letters above the bars denote significant differences between treatments (*p* <0.05).

A similar pattern was also observed for the cauliflower head diameter, with the highest value recorded for the mixture of all fertilizers (T11 and T12, with diameters of 31.24 and 30 cm, respectively) (Figure 1b). Combined treatments (T6 and T7) or mineral-only fertilization (T1) achieved diameters of 28.21, 29.17, and 28.83 cm, respectively, which were significantly higher than those observed for most treatments relying solely on biofertilizer or organic fertilizer. The pattern for treatments that involved only organic or biofertilizer showed average head diameters of 23.84 and 24.75 cm, respectively, with no significant difference from the control. As for the plant height, this indicates a slower release of nutrients from these fertilizers.

The highest results obtained from the fertilizer mixture can be attributed to the positive interaction among them, where the combination balances the rapid nutrient availability from chemical fertilizers with the slow and delayed release from organic and biofertilizers, thereby supporting continuous plant growth and increased height. Different studies^40,41^ reported that integrated fertilization systems can enhance nutrient use efficiency and improve various growth parameters, including plant height.

Mixtures involving mineral fertilizers had a direct effect on increasing plant height and head diameter, especially when used at relatively high proportions (75%) combined with organic or biofertilizer (Figure 1b). Based on these findings, mixtures of mineral, organic, and biofertilizers represent an effective strategy for improving vegetative growth traits, including plant height, particularly in calcareous soil that poses challenges for nutrient availability.

#### 3.1.2 Plant Yield

As for the plant height and diameter, the highest plant yield was achieved with mixtures of all fertilizers with 43.35 and 41.86 tons ha^–1^, with significant differences compared to the control treatment (no fertilization) (30 tons ha^–1^) or treatments that involved an individual fertilizer (Figure 1c). Treatments with the highest amount of conventional mineral fertilizer (75% mineral fertilizer) and organic or biofertilizer (i.e., T6 and T7 with 25% organic and biofertilizers, respectively) also showed a high yield, highlighting the importance of using combined fertilization to enhance the fertilization efficiency and reduce nutrient losses. The highest yield on T11 and T12 treatments can be attributed to a positive interaction between mineral fertilization, fermented cow manure, and biofertilizers. The combination of these sources achieved a balance between the rapid nutrient availability from chemical fertilizers and the slow, sustained release from fermented cow manure and biofertilizers, thus supporting head development and increasing yield weight.

These findings are consistent with those who reported that integrated nutrient management enhances nutrient use efficiency and improves crop productivity.^3,21^ Moreover, high proportions of mineral fertilizer combined with organic or biofertilizer had a direct positive impact on the yield. This aligns with different studies,^3,42–44^ which indicated that biofertilizer application can help in phosphorus mobilization from soil to plants, combined application with organic fertilizers can improve the effectiveness of soil bacterial species and soil biota, and the presence of *Azotobacter* can act as a plant growth promoter.^42^

### 3.2 Total Nitrogen, Phosphorus, and Potassium Content in Cauliflower Heads

The nitrogen content in cauliflower heads was generally significantly higher under all fertilization treatments (conventional mineral fertilizer, organic manure, and biofertilizer) than under the control (T0) (Figure 2a).

**Figure 2:**
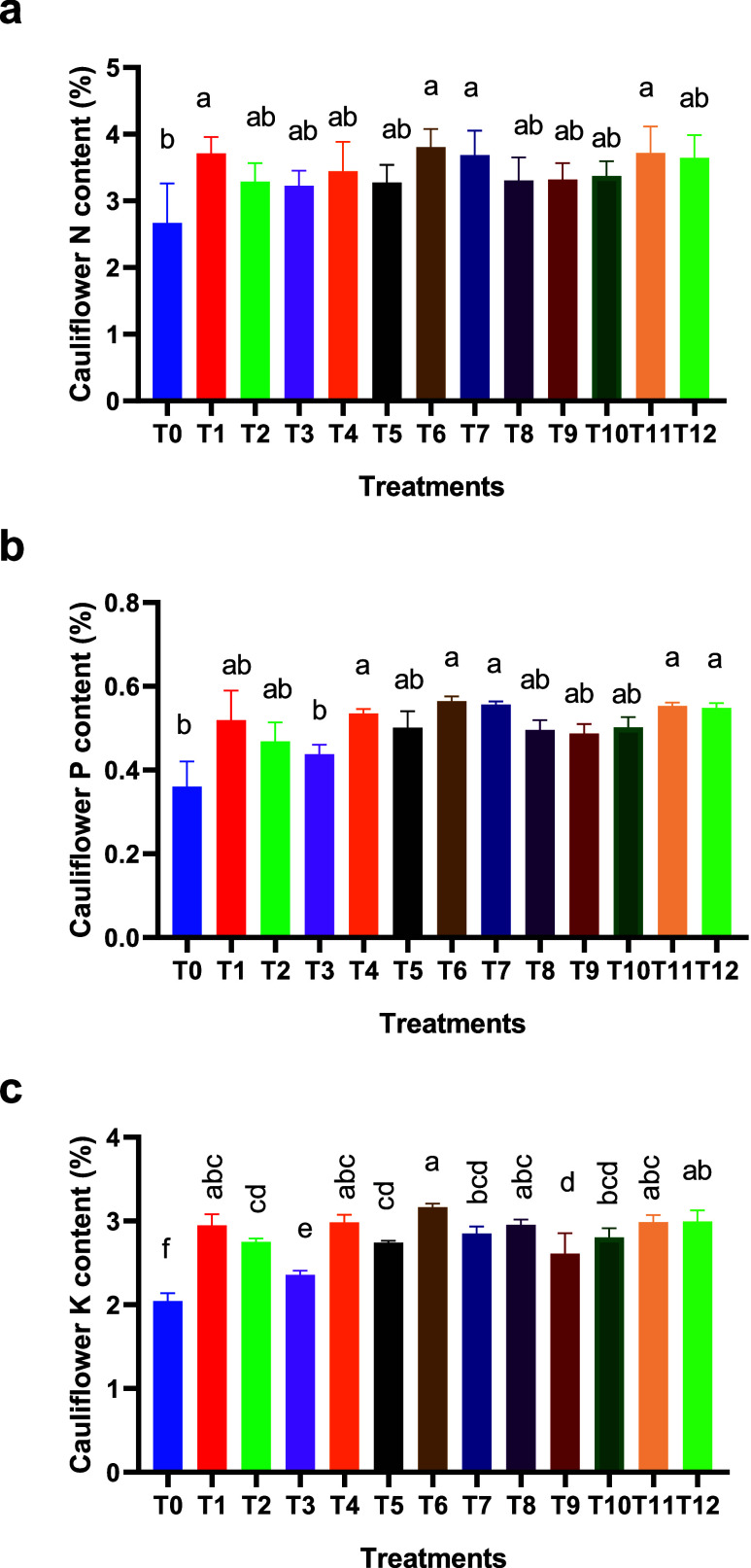
Nitrogen (a), phosphorus (b), and potassium (c) contents in cauliflower heads grown in soils treated with different fertilization treatments. Data represented are mean ± SD (*n* = 3). Letters above bars denote significant differences between treatments (*p* <0.05).

This demonstrated how effective these fertilizers are in increasing the plant’s availability of nutrients, particularly under mixed fertilization (75% mineral fertilizer + 25% fermented cow manure), which had the highest total nitrogen content (3.79%). The nitrogen contents of T8 (25% mineral fertilizer + 75% organic fertilizer), T5 (50% mineral fertilizer + 50% biofertilizer), and T9 (25% mineral fertilizer + 75% biofertilizer) were 3.30%, 3.26%, and 3.28%, respectively. The phosphorus content varied from 0.36 (control treatment) to 0.56% (75% mineral fertilizer + 25% organic fertilizer) (Figure 2b). For T7 (75% mineral fertilizer + 25% biofertilizer), T11 (50% mineral fertilizer + 25% fermented cow manure + 25% biofertilizer), and T12 (33% mineral fertilizer + 33% fermented cow manure + 33% biofertilizer), mixtures performed better than individual applications in nitrogen’s case, suggesting a synergistic effect that improves phosphorus uptake in the plant (Figure 2b). Finally, the potassium content in cauliflower heads ranged from 2.04% in the control treatment (T0) to 3.16% in T6 treatment (75% mineral fertilizer + 25% organic fertilizer), which recorded the highest value (Figure 2c), followed by T12 (33% mineral fertilizer + 33% fermented cow manure + 33% biofertilizer), T4 (50% mineral fertilizer + 50% fermented cow manure), and T11 treatments (50% mineral fertilizer + 25% fermented cow manure + 25% biofertilizer).

These results align with those reported in other studies. In general, the combined application of mineral and organic fertilizers in cauliflower significantly increased the nitrogen concentration in floral heads compared to unfertilized controls.^45,46^ These results also align with those reported,^2^ who found that the combined application of chemical fertilizers and bio-organic fertilizers significantly increased the available phosphorus in soil and improved nutrient uptake and the cauliflower yield. Co-inoculation of phosphate-solubilizing bacteria and fungi enhances phosphorus absorption and plant growth via improved phosphorus cycling in the rhizosphere.^47^ Similarly, Mahmood et al.^48^ also reported similar results for potassium in plants when applying different mixtures of minerals, fermented cow manure, and biofertilizers on the lettuce yield and the potassium content in plants.

### 3.3 Soil Macronutrients

#### 3.3.1 Total Nitrogen and Mineral Nitrogen Contents in Soil

The results of postharvest soil analysis after plant growth showed a significant increase in total nitrogen content in all fertilization treatments compared to the control treatment (no addition) (Figure 3a). The highest nitrogen level was observed in treatment T1 (100% conventional fertilization), reaching 0.54 g kg^–1^, followed by T12 (33% conventional fertilizer, 33% organic fertilizer + 33% biofertilizer) and T11 (50% conventional fertilizer + 25% organic fertilizer + 25% biofertilizer), but without significant differences among these three treatments (Figure 3a). Conversely, treatments relying only organic and/or biofertilizers, such as T9 (50% fermented cow manure + 50% biofertilizer) and T3 (100% biofertilizer), showed lower nitrogen levels (around 0.40 g kg^–1^) (Figure 3a), which suggest that nutrient release from these sources is slower compared to mineral fertilizers, despite their beneficial long-term effects on soil fertility.

**Figure 3:**
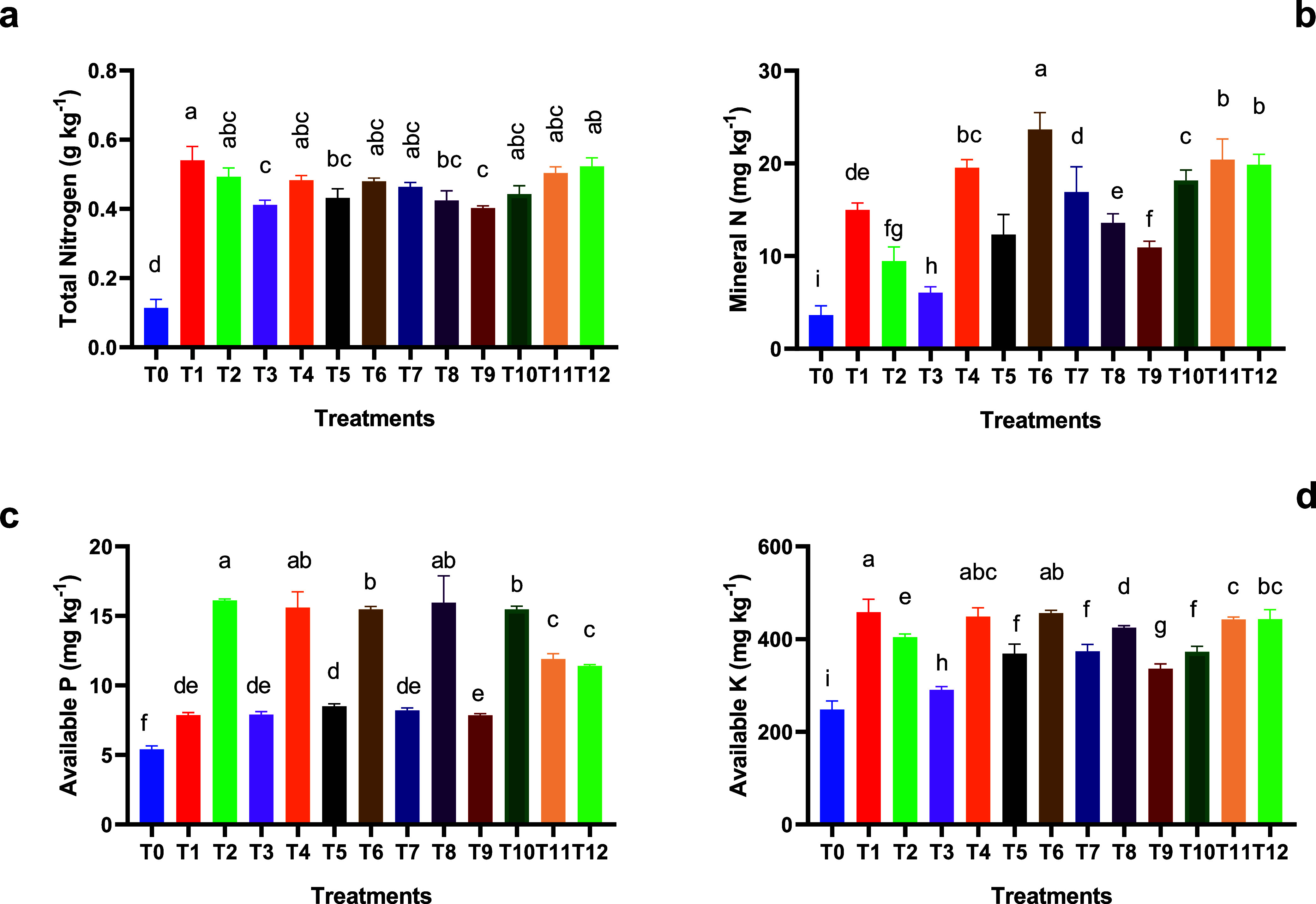
Total Nitrogen (a), mineral N (b), available phosphorus (c), and available potassium (d) in soil treated with different fertilization treatments. Data represented are mean ± SD (*n* = 3). Letters above bars denote significant differences between treatments (*p* <0.05).

Regarding mineral N, the control treatment (T0) had the lowest mean value of soil mineral N (3.60 mg kg^–1^), while the T6 treatment (75% conventional mineral fertilizer + 25% organic fertilizer) exhibited the highest concentration at 23.65 mg kg^–1^, followed by T11, T12, and T4, with mean values over 19.5 mg kg^–1^. Lower values, but higher than those of the control treatment, were observed for 100% application of organic manure (T2) and the biofertilizer (T3) (Figure 3b).

These findings confirm that mineral fertilizers remain the most immediate and effective source of nitrogen in the studied soils. However, the integrated use of minerals, organics, and biofertilizers helps sustain nitrogen availability over time, as observed in the data when mixtures were applied. As expected, increased nitrogen application rates directly enhance the soil nitrogen content.^49^ However, it is also necessary to consider that the fermented cow manure had a low nitrogen content (approximately 2%), which can also explain the differences between conventional and organic fertilizer application. Higher levels of soil mineral nitrogen were consistently obtained from treatments that included mineral fertilizers, either separately or in combination. This was particularly true when low dosages of organic mineral fertilizers were administered. In general, mineral fertilizers offer nitrogen more readily available for plant uptake than organic amendments.^50^

#### 3.3.2 Available Phosphorus and Potassium Content in Soil

The available phosphorus content ranged from 5.40 (no fertilization) to 16.12 mg kg^–1^ (100% organic fertilization). Treatments with mixed balanced mixtures (T4, T8, T10, or T11) also showed an improvement in phosphorus availability (Figure 3c). This suggests the role of organic matter in solubilizing insoluble phosphate compounds and gradually releasing phosphorus, consistent with Abolfazli et al.^51^ for slightly alkaline soils, which highlighted that the incorporation of organic matter can supply phosphorus for plants, releasing available phosphorus over time.

The levels of available potassium in studied soils ranged from 248 (no fertilization) to 458.1 mg kg^–1^ (100% conventional mineral fertilization), with high values under 100% organic fertilization (404 mg kg^–1^) but very low under individual biofertilization (T3) (290 mg kg^–1^), values slightly higher than no fertilization treatment. Fertilization mixtures also showed higher values, although without a clear pattern (Figure 3d). In general, the results highlight the role of organic amendments in improving soil fertility and enhancing the release of macronutrients, such as phosphorus and potassium. These results also emphasize that the combination of mineral and organic fertilizers is one of the most effective strategies to improve potassium availability in soil. Different authors reported similar results to our study. Bader et al.^52^ noted that the addition of organic matter significantly increased available soil potassium, while Li et al.^53^ demonstrated that combining mineral potassium fertilizers with organic residues such as crop straw, which significantly enhanced extractable potassium fractions and improved the overall nutrient dynamics of calcareous soil.

## 4 Conclusion

This study demonstrates that integrating mineral fertilizers with organic and biobased inputs can enhance soil fertility, sustain cauliflower yields, and reduce chemical fertilizer use without significant impacts on the plant yield. It was observed that an optimal ratio of 50% mineral, 25% organic, and 25% biofertilizers produced 43.35 t ha^–1^, increasing the yield by 44% over the control, while reducing chemical fertilizer use by half. However, this study has some limitations, such as single-season data, the absence of microbial assays, and site specificity, which mean that these data should be interpreted cautiously, as they reflect the specific soil conditions, climate, and management practices of the study site. Future studies should conduct multiyear and/or multilocation experiments to mitigate potential single-year effects and investigate the possible influence of different soil properties, particularly pH and organic matter content, as well as analyze plant nutritional values, including micronutrients, soluble sugars, and vitamin content. From a practical perspective, these findings support the gradual transition from strictly mineral-based fertilization to more diversified nutrient management approaches that incorporate organic and biofertilizers.

## Data Availability

All data have been made available within the manuscript.
